# On the Accuracy of Genomic Selection

**DOI:** 10.1371/journal.pone.0156086

**Published:** 2016-06-20

**Authors:** Charles-Elie Rabier, Philippe Barre, Torben Asp, Gilles Charmet, Brigitte Mangin

**Affiliations:** 1 MIAT, Université de Toulouse, INRA, Castanet-Tolosan, France; 2 UR4, INRA, Unité de Recherche Pluridisciplinaire, Prairies et Plantes Fourragères, Lusignan, France; 3 Department of Molecular Biology and Genetics, Aarhus University, Slagelse, Denmark; 4 GDEC, UMR INRA-UBP, Clermont-Ferrand, France; 5 LIPM, Université de Toulouse, INRA, CNRS, Castanet-Tolosan, France; Nanjing Forestry University, CHINA

## Abstract

Genomic selection is focused on prediction of breeding values of selection candidates by means of high density of markers. It relies on the assumption that all quantitative trait loci (QTLs) tend to be in strong linkage disequilibrium (LD) with at least one marker. In this context, we present theoretical results regarding the accuracy of genomic selection, i.e., the correlation between predicted and true breeding values. Typically, for individuals (so-called test individuals), breeding values are predicted by means of markers, using marker effects estimated by fitting a ridge regression model to a set of training individuals. We present a theoretical expression for the accuracy; this expression is suitable for any configurations of LD between QTLs and markers. We also introduce a new accuracy proxy that is free of the QTL parameters and easily computable; it outperforms the proxies suggested in the literature, in particular, those based on an estimated effective number of independent loci (*M*_*e*_). The theoretical formula, the new proxy, and existing proxies were compared for simulated data, and the results point to the validity of our approach. The calculations were also illustrated on a new perennial ryegrass set (367 individuals) genotyped for 24,957 single nucleotide polymorphisms (SNPs). In this case, most of the proxies studied yielded similar results because of the lack of markers for coverage of the entire genome (2.7 Gb).

## Introduction

During the last decades, investigators have mainly concentrated on linkage analysis to detect the regions of DNA, so-called quantitative trait loci (QTLs), responsible for quantitative variation. The linkage analysis (LA) is specific because it relies on family data and on pedigrees: segregation of a QTL is studied within a family by means of information related to the family.

In this context, the most popular statistical method for QTL mapping is interval mapping [1]. It involves scanning the genome by means of genetic markers and testing for the presence/absence of a QTL at every location in the genome. The mathematical theory behind this concept has been extensively studied for many years and is now well established [2–4]. According to [5], thousands of QTLs have been detected in plants, animals, and humans by means of interval mapping as a statistical tool. For instance, [6] detected QTLs responsible for a reduction in grain shattering in cultivated rice, and [7] uncovered a QTL responsible for tomato fruit size.

More recently, researchers moved on to genome-wide association studies (GWAS) that are based on unrelated individuals, in contrast to LA. A GWAS allows researchers to analyze individuals without knowing their pedigree. One of the most popular methods relies on the model proposed by [8]. Hundreds of SNP-trait associations have been discovered in humans [9, 10] by means of GWAS: 30 loci are now known to be linked to Crohn’s disease [11], and approximately 40 loci are associated with human height [12, 13].

Nonetheless, both approaches have a drawback: QTLs with very small effects are difficult to detect. Note that most traits of interest can be characterized as complex traits: they are presumably governed by a large number of small-effect QTLs [14–16]. In a large number of studies, the detected QTLs could not explain all genetic variation [17, 18]. It should be noted that this phenomenon explains a part of the so-called missing heritability. Typically, predictions based on selected SNPs have not been reliable.

At present, genomic selection (GS) is focused on prediction of breeding values of selection candidates by means of high density of markers. In contrast to LA and GWAS, the main goal of GS is not to detect QTLs anymore but to predict the future phenotype of young candidates as soon as their DNA has been collected. GS relies on the expectation that some QTLs will be in strong linkage disequilibrium (LD) with at least one marker [19]. From a theoretical point of view, GS differs from LA and GWAS because GS can be viewed as a whole-genome regression analysis [20, 21]: all the marker effects are estimated simultaneously. This way, it accounts for the correlation among SNPs, which is not the case when each SNP is analyzed separately. GS was first applied to animal breeding, especially dairy cattle (see [22]), where this new method has been found to be particularly promising. It was later tested on plants [23], with recent studies on apple [24], sugar beet [25], pea [26], and on inbred lines of rice [27].

A large number of methods can be chosen to make predictions in GS: penalized regression methods (see [28] for a review), Bayesian methods (see for instance [29]), and reproducing kernel Hilbert spaces methods [30, 31] are the most popular tools. Quality of the prediction is usually evaluated by accuracy criteria, such as the correlation between predicted and true breeding values. A large number of formulas for accuracy are now available in the literature. Most of them are inspired by the work of [32] who derived the formulas while relying only on the causal model with fixed effects and assuming independence of causal loci. Later, this work was extended in [33], in order to allow for the presence of a large number of loci (in the genome) that can not be considered independent due to linkage and a fixed genome size. The authors proposed, in particular, to substitute the effective number of independent loci *M*_*e*_ into the original formula of [32]. Subsequently, a large number of research groups built on this concept and proposed different ways of estimating *M*_*e*_. Those methods are either based on the effective population size (e.g., [34, 35]), or on the number of independent tests in association mapping [36]. A comparison among the methods relying on the effective population size is presented in [37].

There are many questions about GS. The choice of the training (TRN) population with respect to the test (TST) population is a hot area of research. This procedure seems to have a strong influence on predictions [38, 39]. In the mixed model framework, [40] proposed an optimization method based on the coefficient of determination. Note that by choosing the most informative individuals to phenotype (i.e., TRN individuals), researchers can use GS as a tool for reducing phenotyping costs. Another area of active research is the long-term behavior of GS [35, 41], for example, the influence of selection as a function of time, or the reliability of the predicted model as a function of time when only the first generation is phenotyped. With the increase in the number of genomic markers because of next-generation sequencing technologies, the question of selecting genomic regions, prior to the learning step has been addressed in simulation studies [42] as well as studies on real-life data [43]. It has been shown that additional biological information can increase GS accuracy.

In the present study, we propose to focus on mathematical properties of the accuracy based on the regression model called random regression best linear unbiased predictor (RRBLUP) or genomic best linear unbiased predictor (GBLUP). This model, initially proposed by [44], is one of the most popular methods for prediction of breeding values. We present here a closed-form expression for the accuracy; this formula is suitable for any configurations of LD between QTLs and markers. Theoretical developments are made possible by analyzing the causal model and prediction model differently; this is generally not the case for investigators working on the mixed model [34, 40], except [45]. Our theoretical formula enables identification of the terms affecting the accuracy in GS, e.g., LD and the link between TRN and TST sets. Besides, with the help of our formula, we can obtain the key result of [32] regarding the accuracy, when we use the same assumptions that those authors used. Another interesting result in our paper is introduction of a new proxy for the accuracy; this proxy is free of the QTL parameters and is easily computable. We show that substituting an estimated effective number of independent loci (*M*_*e*_) into [32]’s formula is not the appropriate way to work with the high dimensional framework. Another quantity is suggested here. This way, our study can be viewed as an answer to the article [37], where the authors expressed doubt about the existing proxies after comparing 145 accuracy values collected from 13 articles on GS.

In the text below, after a description of the mathematical theory, our theoretical results and existing formulas are compared on simulated data. At the end, an illustration of real-life data is presented. We analyzed GS in plant height in perennial ryegrass, using 24,957 SNPs obtained via genotyping by sequencing (GBS) from 367 genotypes.

## Materials and Methods

### The theory

In this section, we assume, without a loss of generality, that coded genotypes at the markers and at QTLs are centered, as well as the phenotypic observations.

#### The causal linear model

The quantitative trait is observed in *n*_TRN_ TRN individuals, and we denote the observations as *Y*_1_, …, *Y*_*n*TRN_. *C* QTLs are present in the genome and have an effect on the quantitative trait. In the text below, *θ*_*j*_ refers to the fixed QTL effect of the *j*-th QTL and *Q*_*i*, *j*_ denotes the corresponding coded genotype for individual *i*. We assume the following causal linear model for the quantitative trait:

$$Y_{i}=\sum_{j=1}^{C}Q_{i,j}\;\theta_{j}+e_{i}\;\left(i=1,\cdots ,n_{\text{TRN}}\right)$$

where $e_{i}∼N(0,\sigma_{e}^{2}$) and $\sigma_{e}^{2}$ denotes the environmental variance.

With matrix and vector notation, this model can be rewritten as

Y=Qθ+e (1)

where ***Q*** is a *n*_TRN_ × *C* matrix, ***Y*** = (*Y*_1_, …, *Y*_*n*TRN_)′, ***θ*** = (*θ*_1_, …, *θ*_*C*_)′, $e∼N(0,\sigma_{e}^{2}I_{n_{\text{TRN}}})$ and ***I***_***n***TRN_ is the identity matrix of size *n*_TRN_.

In the text that follows, ***q***_***i***_ denotes a vector of size *C* × 1 that refers to the “causal genome” of individual *i*.

#### Introducing a TST individual

A supplementary individual, a so-called TST individual (denoted as *n*_TRN + 1_) is genotyped but not phenotyped. With the same notation as in the TRN population, ***q***_***n***TRN + 1_ denotes the genome at QTL locations of individual *n*_TRN + 1_. As a result, the quantitative trait $Y_{n_{\text{TRN}}+1}$ can be expressed as

$$Y_{n_{\text{TRN}}+1}=q_{n_{\text{TRN}}+1}^{\prime }\theta +e_{n_{\text{TRN}}+1}$$

where $e_{n_{\text{TRN}}+1}∼N(0,\sigma_{e}^{2}$). Next, ***q***_***n***TRN + 1_ will be considered random. Recall that ***θ*** is fixed.

#### Accuracy

In GS, we are interested in predicting either the genotypic value $q_{n_{\text{TRN}}+1}^{\prime }\theta$, or the phenotypic value *Y*_*n*TRN + 1_. In both cases, a predictor ${\hat{Y}}_{n_{\text{TRN}}+1}$ is constructed by means of a prediction model developed through learning on *n*_TRN_ TRN individuals. Then, the quality of the prediction is evaluated according to some accuracy criteria. In particular, the phenotypic accuracy, *ρ*, and the genotypic accuracy, $\tilde{\rho }$, are defined as follows:

$$\rho =\frac{\text{Cov}\left({\hat{Y}}_{n_{\text{TRN}}+1},\;Y_{n_{\text{TRN}}+1}\right)}{\sqrt{\text{Var}\left({\hat{Y}}_{n_{\text{TRN}}+1}\right)\text{Var}\left(Y_{n_{\text{TRN}}+1}\right)}}\;\text{,}\;\tilde{\rho }=\frac{\text{Cov}\left({\hat{Y}}_{n_{\text{TRN}}+1},\;q_{n_{\text{TRN}}+1}^{\prime }\theta \right)}{\sqrt{\text{Var}\left({\hat{Y}}_{n_{\text{TRN}}+1}\right)\text{Var}\left(q_{n_{\text{TRN}}+1}^{\prime }\theta \right)}}\;\text{.}$$ (2)

These two types of accuracy are linked by the relation $\rho /\tilde{\rho }=h$, where *h* is the square root of the heritability of the trait:

$$h^{2}=\frac{\theta^{\prime }\text{Var}\left(q_{n_{\text{TRN}}+1}\right)\theta }{\theta^{\prime }\text{Var}\left(q_{n_{\text{TRN}}+1}\right)\theta +\text{Var}\left(e_{n_{\text{TRN}}+1}\right)}\;\text{.}$$ (3)

Next, we set $\sigma_{G}^{2}\;=\;\theta^{\prime }\text{Var}\left(q_{n\text{TRN}+1}\right)\theta$, and as a consequence, we have the relationship $h^{2}=\sigma_{G}^{2}/(\sigma_{G}^{2}+\sigma_{e}^{2})$. Depending on a research group, investigators focus either on phenotypic accuracy *ρ* (e.g., [46]), or on genotypic accuracy $\tilde{\rho }$ (e.g., [32, 33]).

#### The oracle situation

Suppose this situation denotes the settings where the QTL locations and their effects are known. Then, the natural predictor, ${\hat{Y}}_{n_{\text{TRN}}+1}$, of the quantity *Y*_*n*TRN + 1_ is

$${\hat{Y}}_{n_{\text{TRN}}+1}=q_{n_{\text{TRN}}+1}^{\prime }\theta \;\text{.}$$

As a result, the oracle accuracies, so-called *ρ*_oracle_ and ${\tilde{\rho }}_{\text{oracle}}$ for phenotypic and genotypic accuracy, satisfy the following equations:

$$\rho_{\text{oracle}}=\frac{\text{Cov}\left(q_{n_{\text{TRN}}+1}^{\prime }\theta ,\;Y_{n_{\text{TRN}}+1}\right)}{\sqrt{\text{Var}\left(q_{n_{\text{TRN}}+1}^{\prime }\theta \right)\text{Var}\left(Y_{n_{\text{TRN}}+1}\right)}}=h\;\text{and}\;{\tilde{\rho }}_{\text{oracle}}=1\;\text{.}$$

#### A marker-based model

In practice, the QTL effects and their locations are typically unknown. As a consequence, the prediction is based on information from *p* genetic markers located in the genome. Suppose ***X*** denotes the TRN incidence matrix of size *n*_TRN_ × *p*. The TRN marker-based model is typically the following random effects model:

Y=Xβ+ε

where ***Y*** = (*Y*_1_, …, *Y*_*n*TRN_)′, $\beta =(\beta_{1},\ldots ,\beta_{p})\prime ∼N\left(0,\sigma_{\beta }^{2}I_{p}\right)$, $\varepsilon ∼N(0,\sigma_{\varepsilon }^{2}I_{n_{\text{TRN}}})$. This setting allows to work with the high dimensional framework, i.e. the situation where *p* > *n*_TRN_. $\sigma_{\beta }^{2}$ and $\sigma_{\varepsilon }^{2}$ denote respectively the variance of the marker effects and the residual variance.

By the same token, ***x***_***i***_ is a vector of size *p* × 1 that refers to genomic markers of individual *i*. Recall that ***q***_***i***_ represents the “causal genome” of individual *i*. Besides, in the text that follows, we use the notation *X*_*i*,*j*_ for the coded genotype of individual *i* at the *j*-th marker.

This model was initially proposed by [44]. In the literature, it is known as GBLUP or RRBLUP. As a consequence, the estimated effects of SNPs are

$$\hat{\beta }=E\left(\beta |Y,X\right)={\left(X^{\prime }X+\lambda I_{p}\right)}^{-1}X^{\prime }Y\;\text{where}\;\lambda =\sigma_{\varepsilon }^{2}/\sigma_{\beta }^{2}\text{.}$$

Suppose ***x***_***n***TRN + 1_ denotes the random variable corresponding to the genomic markers of the TST individual. Then, the prediction is

$$\begin{array}{lll} {\hat{Y}}_{n_{\text{TRN}}+1}=x_{n_{\text{TRN}}+1}^{\prime }\hat{\beta } & = & x_{n_{\text{TRN}}+1}^{\prime }{\left(X^{\prime }X+\lambda I_{p}\right)}^{-1}X^{\prime }Y\\ & = & x_{n_{\text{TRN}}+1}^{\prime }X^{\prime }V^{-1}Y\text{ where }V=XX^{\prime }+\lambda I_{n_{\text{TRN}}}\;. \end{array}$$ (4)

The RRBLUP model is also called ridge regression and the parameter λ is viewed as a regularization parameter (see for instance [47]).

The main result of this paper is the following. Recall that ***θ*** is fixed, and that ***q***_***n***TRN + 1_ and ***x***_***n***TRN + 1_ are random. Conditionally on both the TRN incidence matrix ***X***, and the TRN causal matrix ***Q***, the phenotypic accuracy is

$$\rho_{\text{RR}}=\frac{\theta^{\prime }\;E\left(q_{n_{\text{TRN}}+1}x_{n_{\text{TRN}}+1}^{\prime }\right)X^{\prime }V^{-1}Q\theta }{{\left(\sigma_{e}^{2}\;E\left({\left\|x_{n_{\text{TRN}}+1}^{\prime }X^{\prime }V^{-1}\right\|}^{2}\right)\;+\;\theta^{\prime }Q^{\prime }V^{-1}X\text{Var}\left(x_{n_{\text{TRN}}+1}\right)X^{\prime }V^{-1}Q\theta \right)}^{1/2}{\left(\sigma_{G}^{2}+\sigma_{e}^{2}\right)}^{1/2}}$$ (5)

where ‖.‖ is the *L*^2^ norm, and Var(***x***_***n***TRN + 1_) is the covariance matrix of size *p* × *p*. The proof is provided in S1 Text.

Finally, we want to emphasize that this closed-form expression for the accuracy was derived without any assumptions on QTL locations and marker locations. In other words, the formula deals with the configuration where QTLs match a few genetic markers as well as the configuration where QTLs are not located on markers.

#### A new proxy (QTLs in perfect LD with some markers)

Let us assume now that the *C* QTLs are located exactly on genetic markers, but at the same time, let us allow the number of genetic markers to be much larger than the number of QTLs (i.e., *p* > > *C*). Suppose that

$$x_{n_{\text{TRN}}+1}^{\prime }X^{\prime }V^{-1}Q\theta =q_{n_{\text{TRN}}+1}^{\prime }\theta \;\text{.}$$

This is an ideal situation where each QTL is in perfect LD with its associated marker, with respect to the ***V*** ^**−1**^ matrix. Besides, each QTL is in linkage equilibrium with other markers, with respect to the ***V*** ^**−1**^ matrix. This assumption is appropriate in a random mating population (with a large number of individuals), evolving during a large number of generations because the LD decreases at an exponential rate (e.g., [48, 49]).

Then, according to formula (5) and the proof provided in S1 Text, the accuracy becomes

$$\rho_{\text{pLD}}=h\;\sqrt{\frac{h^{2}/\left(1-h^{2}\right)}{E\left({\left\|x_{n_{\text{TRN}}+1}^{\prime }X^{\prime }V^{-1}\right\|}^{2}\right)+\frac{h^{2}}{1-h^{2}}}}\;\text{.}$$ (6)

Contrary to the general accuracy presented in formula (5), this accuracy can be computed easily because it depends on known or estimable quantities: the heritability of the trait is usually known, and the expectation on the denominator can be estimated using the empirical mean in the TST sample. Finally, the tuning parameter λ that is present in the expression for ***V***, can be estimated by several statistical methods. In this paper, we used Restricted Maximum Likelihood (REML) [50] or deduced λ from the heritability.

#### The link with the work of [32]

In [32], a seminal formula for the accuracy is presented. We would like to show here that with the help of our general formula (5), we can obtain this previously published result, if we use the same assumptions that those authors used. In particular, [32] assumed that the QTL locations are known and that each QTL is in perfect LD with its associated marker. The formula was obtained by performing regression analysis of the trait on each QTL separately; this approach is equivalent to assuming that ***Q*′*Q*** is diagonal and thus invertible. Even the case *C* >> *n*_TRN_ can be analyzed because of this above assumption. Then, the estimated QTL effects and the prediction are

$$\hat{\beta }={\left(Q^{\prime }Q\right)}^{-1}Q^{\prime }Y\;\text{,}\;{\hat{Y}}_{n_{\text{TRN}}+1}=q_{n_{\text{TRN}}+1}^{\prime }{\left(Q^{\prime }Q\right)}^{-1}Q^{\prime }Y\;\text{.}$$

Note that $\hat{\beta }$ is obtained by assuming that λ = 0 in formula (4). In this context, according to our general formula (5) and calculations shown in S1 Text, the accuracies are the following:

$$\tilde{\rho }=\sqrt{\frac{h^{2}/\left(1-h^{2}\right)}{\frac{C}{n_{\text{TRN}}}\;+\;\frac{h^{2}}{1-h^{2}}}}\;\text{,}\;\rho =h\;\sqrt{\frac{h^{2}/\left(1-h^{2}\right)}{\frac{C}{n_{\text{TRN}}}\;+\;\frac{h^{2}}{1-h^{2}}}}\;\text{.}$$ (7)

This expression for $\tilde{\rho }$ is suitable for any values of $\sigma_{G}^{2}$ and $\sigma_{e}^{2}$. This is not the case in [32] because those authors analyzed the case $\sigma_{G}^{2}+\sigma_{e}^{2}=1$ and used the approximation $\sigma_{e}^{2}=1$. We refer readers to S1 Text for more details.

### Simulation study

In order to verify the validity of our theoretical results, a simulation study was performed.

#### Simulated data

Genomic data were generated by means of the hypred R package [51]. Populations were simulated by random mating between haploid individuals, during (a) 30, (b) 50, or (c) 70 generations. Recombination was modeled according to Haldane [52]. Mutations were not taken into consideration. In generation zero, two haploid founder lines were crossed. These two lines were completely different genetically. Generation 1 consisted of (a) 400 or (b) 800 haploid offspring of these two founders. After that, the population kept evolving by random mating with a constant size at each generation and no overlapping generation. This type of simulation mimics recombinant inbred line (RIL) or double haploid (DH) evolving populations. In the final generation, 2 individuals were randomly selected, and 100 (resp. 200) full sibs were generated under the 400 (resp. 800) offspring scenario, in order to get some closely related individuals.

The focus was on one chromosome of length 1 Morgan. We considered 4 different densities of genetic markers equally spaced on the chromosome: (a) 100, (b) 1,000, (c) 5,000, or (d) 10,000 SNPs. We considered two configurations for the phenotypic model: (a) 2 QTLs located at 3cM and 80cM with effects +1 and −2, respectively, or (b) 100 QTLs located every centimorgan, with the same effect +0.15. The environmental variance $\sigma_{e}^{2}$ was set to 1. Table 1 shows the estimated heritabilities corresponding to the different scenarios studied. These heritabilities are based on the overall population (TRN+TST). Indeed, although a few closely related individuals were present in the TRN set, we did not observe a noticeable change in terms of heritability between the TST set and the TRN set.

**Table 1:** Estimated heritability (*h*^2^) as a function of the simulation setup.

Nb QTLs	Nb Generations	*n*_TRN_	*h*^2^
2	30	500	0.54
		1,000	0.53
	50	500	0.53
		1,000	0.53
	70	500	0.51
		1,000	0.49
100	30	500	0.75
		1,000	0.77
	50	500	0.65
		1,000	0.69
	70	500	0.57
		1,000	0.61

The average of 100 replicates. In each sample, heritability estimated using the estimator $\hat{\text{Var}(q_{i}^{\prime }\theta )}/(\hat{\text{Var}(q_{i}^{\prime }\theta )}+1)$ based on the overall population (*n*_TRN_ TRN + 100 TST).$\hat{\text{Var}}$ denotes empirical variance.

Genetic markers without polymorphism were filtered out. Besides, identical SNPs along the chromosome were also filtered out; we kept only the first occurrence of that SNP on the chromosome.

A set of either (a) 500 or (b) 1,000 TRN individuals was used for the learning step. Note that in both cases, the TRN set included the full sibs: among the 500 (resp. 1,000) TRN, 100 (resp. 200) were full sibs. The prediction model was evaluated on 100 TST (in all cases), that were produced in the last generation.

In the text that follows, an “architecture” is a fixed number of: (a) SNPs; (b) generations; (c) QTL numbers, effects, and locations; (d) TRN individuals. A total of 100 replicates were generated according to a given architecture. Table 2 shows a summary of the different configurations studied, and Table 3 provides the number of remaining markers after filtering. As in [53], the QTL locations did not vary across replicates. Nonetheless, contrary to that article, the QTL effects always had the same values here.

**Table 2:** The different configurations studied.

Nb markers	100 / 1,000 / 5,000 / 10,000
Nb Generations	30 / 50 / 70
Nb QTLs	2 / 100
*n*_TRN_	500 / 1,000

**Table 3:** The average number of markers after filtering (based on 100 replicates).

Nb generations	Nb Markers	*n*_TRN_	Nb Markers after filtering
30	100	500	100
		1,000	100
	1,000	500	766.51
		1,000	929.22
	5,000	500	1,262
		1,000	2,066.17
	10,000	500	1,353
		1,000	2,345.53
50	100	500	100
		1,000	100
	1,000	500	801.49
		1,000	950.08
	5,000	500	1,392.73
		1,000	2,267.54
	10,000	500	1,518
		1,000	2,609.32
70	100	500	99.98
		1,000	100
	1,000	500	812.37
		1,000	950.68
	5,000	500	1,451.6
		1,000	2,380.81
	10,000	500	1,591
		1,000	2,781.81

#### Regularization parameter λ

For each replicate, predicted phenotypes of the TST dataset were obtained by RRBLUP. The regularization parameter λ was estimated in two ways. The first method relies on variance components estimated by REML. Then, the corresponding regularization parameter called λ_*REML*_ is

$$\lambda_{REML}={\hat{\sigma }}_{\varepsilon }^{2}/{\hat{\sigma }}_{\beta }^{2}$$

where ${\hat{\sigma }}_{\varepsilon }^{2}$ and ${\hat{\sigma }}_{\beta }^{2}$ denote respectively the estimates of the environmental variance $\sigma_{\varepsilon }^{2}$ and the variance $\sigma_{\beta }^{2}$ of each SNP effect. The rrBLUP R package and in particular its function kin.blup were used to compute these variance components.

The second method relies on the heritability of the quantitative trait assuming that the genetic variance is spread out uniformly across all the genetic markers. Then, the tuning parameter called λ_*h*^2^_ is defined as follows:

$$\lambda_{h^{2}}=\frac{1-h^{2}}{n_{\text{TRN}}\;h^{2}}\;\sum_{j=1}^{\tilde{p}}\sum_{i=1}^{n_{\text{TRN}}}X_{i,j}^{2}$$

where $\tilde{p}$ is the number of markers after filtering, and *h*^2^ is the estimated heritability given in Table 1.

#### Empirical accuracy and Theoretical accuracy

In the text below, *n*_TST_ denotes the number of TST individuals, and ***x***_***n***TRN_ + ***i*** means the genomic markers of the *i*-th TST individual. In order to compute the so-called Theoretical accuracy, introduced in formula (5), we used the following estimators:

$$\begin{array}{l} \frac{1}{n_{\text{TST}}}\sum_{i=1}^{n_{\text{TST}}}{\left\|x_{n_{\text{TRN}}+i}^{\prime }X^{\prime }V^{-1}\right\|}^{2}\;,\;\theta^{\prime }\;\left(\frac{1}{n_{\text{TST}}}\sum_{i=1}^{n_{\text{TST}}}q_{n_{\text{TRN}}+i}\;x_{n_{\text{TRN}}+i}^{\prime }\right)X^{\prime }V^{-1}Q\theta ,\\ \frac{1}{n_{\text{TST}}}\;\left[x_{n_{\text{TRN}}+1},\ldots ,x_{n_{\text{TRN}}+n_{\text{TST}}}\right]\times {\left[x_{n_{\text{TRN}}+1},\ldots ,x_{n_{\text{TRN}}+n_{\text{TST}}}\right]}^{\prime }\;,\\ \frac{1}{n_{\text{TST}}}\sum_{i=1}^{n_{\text{TST}}}{\left(q_{n_{\text{TRN}}+i}^{\prime }\theta -\frac{1}{n_{\text{TST}}}\sum_{i=1}^{n_{\text{TST}}}q_{n_{\text{TRN}}+i}^{\prime }\theta \right)}^{2}\;, \end{array}$$

to estimate the quantities $E\left({\left\|x_{n\text{TRN}+1}^{\prime }X^{\prime }V^{-1}\right\|}^{2}\right)$, $\theta^{\prime }\;E(q_{n_{\text{TRN}}+1}x_{n_{\text{TRN}}+1}^{\prime })X^{\prime }V^{-1}Q\theta$, Var(***x***_***n***TRN + 1_), and $\sigma_{G}^{2}$, respectively. Besides, the true value was used for the environmental variance, i.e., $\sigma_{e}^{2}=1$. The empirical accuracy was computed in the R software, with the empirical correlation between the predicted values and the true values.

#### Mean accuracy on replicates and the TRN incidence matrix

Recall that our theoretical result in formula (5) was obtained conditionally on the TRN incidence matrix ***X*** and conditionally on the TRN causal matrix ***Q***. In most of the simulation results presented in this paper, ***X*** and ***Q*** are different across replicates. Indeed, for each replicate, a new population was generated by random mating according to the given architecture. The accuracy was computed according to formula (5) on each replicate, and finally, the mean accuracy was calculated on the 100 replicates. As a consequence, the focus was on a mean accuracy corresponding to a given architecture.

On the other hand, we also analyzed the case where ***X*** and ***Q*** do not vary across replicates. In this case, ***X*** and ***Q*** were obtained by generating only one TRN population associated with a given architecture. Only the TST incidence matrix was allowed to change across replicates. In particular, TST individuals were regenerated by random mating between individuals from the penultimate generation. New phenotypes (TRN+TST) were regenerated for every replicate, and as previously, the mean accuracy on the 100 replicates was computed.

#### The effective number of segments *M*_*e*_

Most of the published proxies for the accuracy use the so-called effective number of independent loci (*M*_*e*_). We focused on the three following representations of *M*_*e*_:

$$M_{e1}=\frac{2N_{e}L}{\log(4N_{e}l)}\;\text{,}\;M_{e2}=\frac{2N_{e}L}{\log(2N_{e}l)}\;\text{,}\;M_{e3}=\frac{2N_{e}L}{\log(N_{e}l)}$$

where *L*, *l*, and *N*_*e*_ denote the genome length, average chromosome length, and effective population size respectively. *M*_*e*1_ was proposed by [35], whereas *M*_*e*2_ and *M*_*e*3_ are from [34]. In our simulation study, *N*_*e*_ was estimated using theoretical results of [54]. In particular, the LD was computed between all SNPs (in the TRN incidence matrix), and the estimated *N*_*e*_ was the least-squares estimate of the fitted nonlinear model (cf. S2 Text). Another approach developped to handle the issue of multiple testing in association mapping studies [36] was also considered. Later, this method was applied to GS (e.g. [55]). The drawback of this method is that it requires that *p* < *n*_TRN_ + *n*_TST_. To overcome this problem, the chromosome was split into 2 parts for the 1,000 SNPs scenario, and into 3 parts when 5,000 SNPs or 10,000 SNPs were analyzed. After that, the overall *M*_*e*_ was obtained by summing up the numbers of independent tests obtained separately for each part.

### Real data study on perennial ryegrass

Our plant material belongs to the perennial ryegrass species (*Lolium perenne* L.), a diploid species (2n = 14) with a haploid genome size of 2.7 Gb [56]. This genome size was estimated by flow cytometry in picograms and transformed in to the number of bases assuming 978 Mb/pg [57]. *Lolium perenne* L. is a highly heterozygous species with strong inbreeding depression and a self-incompatibility system. Besides, the varieties are synthetics. The population was provided by the private breeding company Gie GRASS. The dataset consisted of 367 genotypes obtained after the multiplication by intercrossing of 12 genotypes during three generations. Moreover, the 12 genotypes were obtained from pair-crosses involving 8 different genotypes. Seeds were sown in individual pots in the first week of August 2013. They were cut regularly to promote tillering and were cloned. On April 16th, 2014, four clones per genotype were planted in the field at INRA Lusignan France (43°36’55.59”N; 4° 0’36.59”E) in randomized block design. On August 21st, 2014, the plants were cut at approximately 5 cm and plant height was measured immediately with a ruler. Plant height was remeasured on August 28th, 2014. The plant growth rate was calculated as the difference in plant height between the two dates divided by the number of growing degree days, with the base temperature of zero (138.5°C.days).

Molecular data were obtained by GBS following the same protocol as in [58]. DNA was extracted from 50 mg of dried leaves by the protocol described in [59]. PstI was used for complexity reduction. The sequencing was performed by means of the Hiseq 2500 (Illumina; pair-end 2 × 150, but only one pair was used for analysis). Scythe (https://github.com/vsbuffalo/scythe) was used to demultiplex the GBS raw reads and to trim adaptor contamination, using the prior contamination rate set to 0.40. Sickle (https://github.com/najoshi/sickle) was used to quality-trim the demultiplexed reads using the parameters -q 20 -l 40. The demultiplexed and quality trimmed reads were aligned against a draft assembly of the perennial ryegrass genome (48,415 scaffolds) in the BWA aln software [60]. The resulting BAM files were further processed using the Genome Analysis Toolkit (GATK) version 2.7-4 [61]. Variant calling was performed by means of GATK’s UnifiedGenotyper, and high-quality SNPs were extracted. The resulting SNPs were quality-filtered according to several criteria, for example, only variants with a quality score higher than 30 were retained. A total of 24,957 SNPs with the minimum allele frequency of 5% were scored. Plink [62] was used to calculate the Pearson coefficient of correlation between pairs of SNPs belonging to the same scaffold. Phenotypic and SNP data are available at doi:10.5061/dryad.jb17n.

## Results

### Simulated data

This section starts by considering QTLs in perfect LD with some markers. Later, the case of imperfect LD is also reviewed. We studied successively, with the help of simulated data, (a) reliability of the Theoretical accuracy from formula (5), (b) sensitivity to the regularization parameter λ, (c) the effects of a fixed TRN incidence matrix, (d) the pertinence of the proxy suggested by formula (6), and (e) a substitute for the effective number of segments. Note that in the following text, the TRN incidence matrix varies across replicates, unless stated otherwise.

#### Empirical accuracy versus Theoretical accuracy

**Fig 1:**
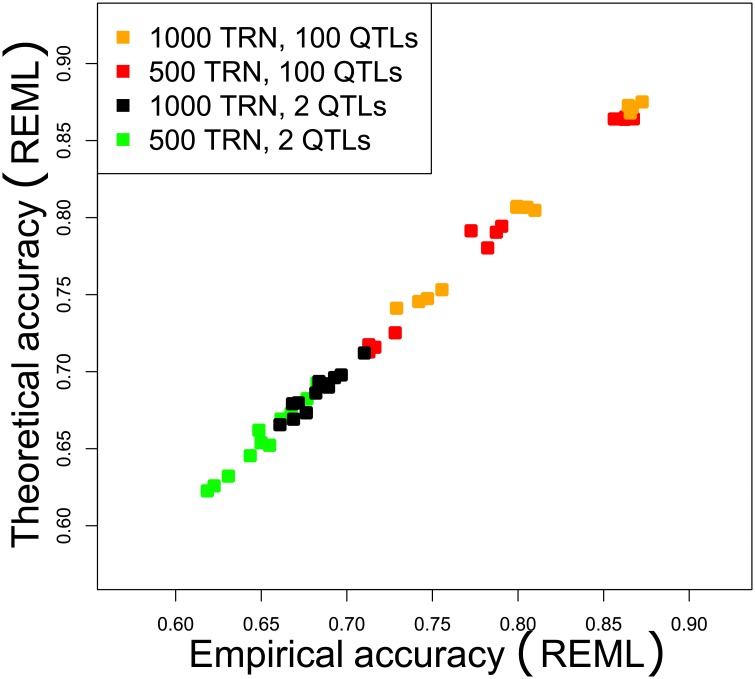
Comparison between the Theoretical accuracy and the Empirical accuracy, as a function of the number of TRN individuals and the number of QTLs. Tuning parameter λ was estimated by REML in both cases.

Fig 1 shows a comparison between the Empirical accuracy and Theoretical accuracy. The tuning parameter λ was estimated by REML in both cases. Each point on the graph corresponds to mean accuracy (based on 100 replicates) associated with a given architecture.

According to the figure, the Theoretical accuracy matched the Empirical accuracy regardless of the architecture being considered. Besides, readers can see that the accuracy increased with the number of QTLs because the heritability increased. As expected, for given numbers of QTLs, generations, and markers, the greater the number of TRN individuals, the higher the accuracy was. For instance, when we considered 50 generations, 2 QTLs and 10K SNPs, the Theoretical Accuracy was estimated to be 0.65 for *n*_TRN_ = 500 and 0.68 for *n*_TRN_ = 1,000, although the heritability was the same.

To complete our simulation study, it is worth to consider the case of a mixture between major genes and multiple small QTLs which mimics probably better the common architecture for a lot of traits. This type of architecture was also used to investigate a larger range of heritability. So, we generated two large QTLs located at 3cM and 80cM, and 98 small QTLs located every centimorgan (except at 3cM and 80cM). We considered three scenarios: (a) large QTLs with effects +0.5 and −0.6, small QTLs with the same effect +0.07, (b) large QTLs with effects +1 and −0.7, small QTLs with the same effect +0.1, (c) large QTLs with effects +2 and −2, small QTLs with the same effect +0.1. In all cases, we focused on the configuration 1,000 SNPs, 500 TRN individuals, and 50 generations. The heritabilities associated to the different scenarios were: (a) *h*^2^ = 0.34, (b) *h*^2^ = 0.54, (c) *h*^2^ = 0.71. According to our simulated data, the Theoretical accuracy matched exactly the Empirical accuracy for scenario (a) (0.52), and scenario (b) (0.68). A very good agreement was also observed for scenario (c): the Empirical accuracy was found to be equal to 0.80, whereas the Theoretical accuracy took the value 0.79.

#### Tuning parameter λ

**Fig 2:**
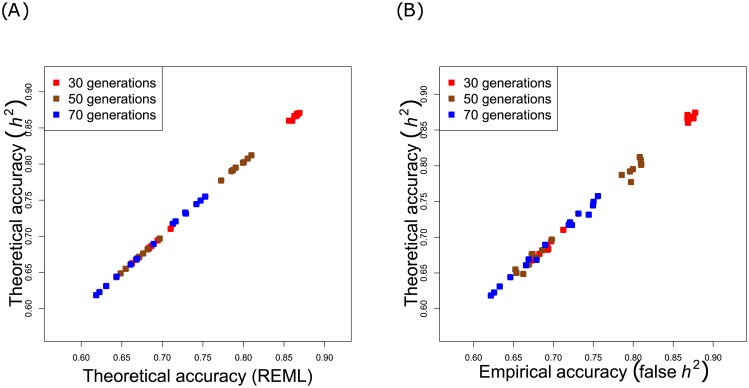
Comparison between the Theoretical accuracies and Empirical accuracies as a function of the tuning parameter λ, and as a function of the number of generations considered. λ was either estimated by REML, or based on either heritability *h*^2^ or on false heritability.

Fig 2 shows analysis of sensitivity of the Theoretical and Empirical accuracies to the regularization parameter λ. We focused on two ways of estimating λ: one used REML, whereas the second one relied on heritability of the trait. According to Fig 2A, the Theoretical accuracy remained unchanged regardless of the method chosen for estimating λ. Because in practice, only approximated heritability is known to geneticists, we considered also the case where the tuning parameter was based on wrongly inferred heritability (90% of the true value). According to Fig 2B, the accuracy did not deteriorate: there was still good agreement between an Empirical accuracy based on a false heritability, and a Theoretical accuracy dependent on the true quantity.

#### QTLs in imperfect LD with some markers

**Fig 3:**
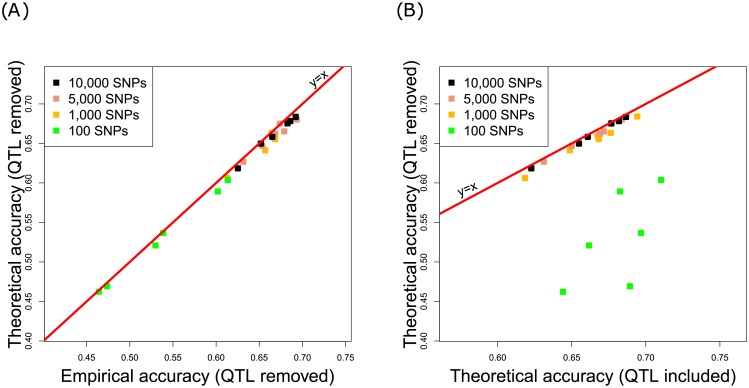
Theoretical accuracies and Empirical accuracies, as a function of the density of markers, and depending on whether the causal SNP was observed. The focus was on the 2 QTL scenario. “QTL removed” means the configuration where the causal SNP was not observed in the TRN and TST populations, whereas “QTL included” means opposite. In all cases, the tuning parameter λ was based on the heritability.

Because our previous analysis implied that QTLs were in perfect LD with some markers, here, we analyze the case of imperfect LD. To mimic imperfect LD, the causal SNPs were unobserved in the TRN and TST populations. Fig 3A focuses on the 2 QTL scenario, and highlights the fact that our theoretical formula is also suitable under imperfect LD. Indeed, for simulated data without the causal SNPs in the marker-based model, the Theoretical accuracy matched the Empirical accuracy for all the different architectures. We also studied the effects of the presence/absence of the causal SNPs in the marker-based model as a function of marker density (Fig 3B). Readers can notice that the Theoretical accuracy was not affected by the absence of the causal SNPs, provided that the density of markers remained high (at least 1,000 markers). As explained in [63], on a dense map, each QTL tends to be in perfect LD with at least one SNP. In contrast, when the density of markers is low, the Theoretical accuracy decreases due to the lack of markers. For instance, according to Fig 3B, when 100 SNPs and 500 TRN individuals were considered, the accuracy decreased respectively from 0.68 to 0.59, from 0.66 to 0.52, or from 0.64 to 0.46, when the population evolved during 30 generations, 50 generations and 70 generations respectively.

#### Only one TRN incidence matrix

**Fig 4:**
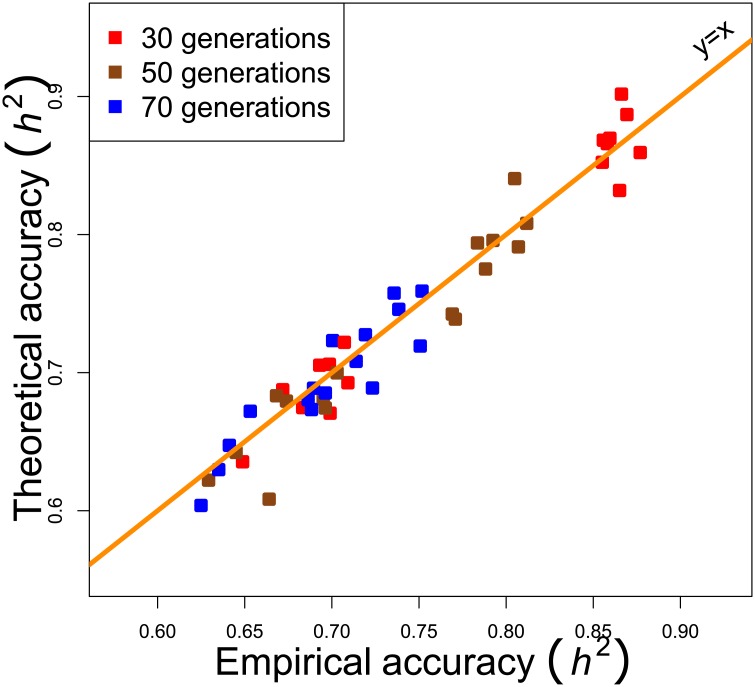
Comparison between the Theoretical accuracy and the Empirical accuracy, as a function of the number of generations. For a given architecture, the TRN incidence matrix did not vary across replicates. The tuning parameter λ was based on the heritability.

Fig 4 shows analysis of the case where the TRN incidence matrix ***X*** and the TRN causal matrix ***Q*** did not vary across replicates (for a given architecture). Readers can see that the Theoretical accuracy and the Empirical accuracy were still a good match. It is noticeable, however, that there was more variability than when ***X*** and ***Q*** varied across replicates.

#### New proxy vs existing proxies

In order to predict the accuracy in genomic selection, most of the methods are based on the original formula of [32]. They consist of replacing the number of independent QTLs by the effective number of independent loci *M*_*e*_. In most cases, *M*_*e*_ is computed according to assumptions of population genetics [34, 35]. Note that *M*_*e*_ can also be computed by inferring the number of independent tests in association mapping studies [36].

**Fig 5:**
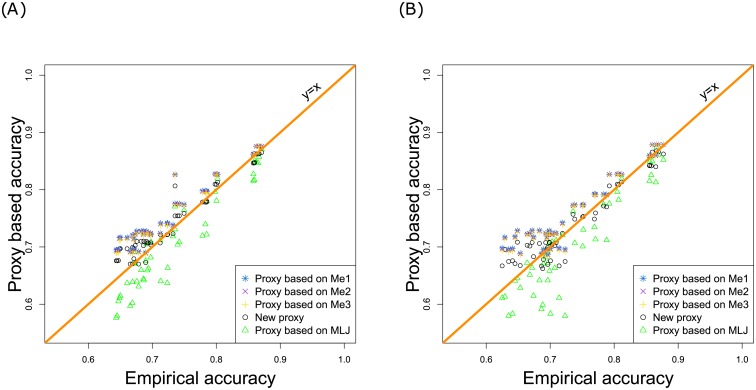
Performances of 5 proxies as a function of the Empirical accuracy. (A) For a given architecture, the TRN incidence matrix varied across replicates. (B) For a given architecture, the TRN incidence matrix did not vary.

In this context, Fig 5 shows a comparison of performance of five different proxies in terms of the accuracy. Three of these proxies, the ones based on *M*_*e*1_, *M*_*e*2_, and *M*_*e*3_, rely on the effective population size, whereas the fourth, an *M*_*LJ*_-based proxy, comes from association studies. The fifth proxy is the one suggested in this paper (formula 6). In Fig 5A, the TRN incidence matrix ***X*** varies across replicates, whereas it is fixed in Fig 5B. In both cases, we can notice that the proxy based on *M*_*LJ*_ underestimated the Empirical accuracy. Table 4 shows the mean squared errors (MSE) corresponding to each method. As expected, the Theoretical accuracy yielded the best performances. Recall that it cannot be computed in practice because it depends on unknown quantities: the QTL effects and their locations. Furthermore, our new proxy outperformed the existing proxies. In comparison with the MSE corresponding to the best proxy (the *M*_*e*3_-based one), our proxy MSE were 2.3- and 1.8-fold smaller, respectively when ***X*** varied and when ***X*** did not vary across replicates. Note also that for each method, the MSE was smaller when ***X*** varied than when ***X*** was fixed (Fig 5).

**Table 4:** Mean squared error (with respect to the Empirical accuracy) corresponding to 5 proxies. The MSE corresponding to the Theoretical accuracy is also shown (λ is based on the heritability). $\text{MSE}=\sum_{a=1}^{48}{\left({\text{AccP}}_{a}-{\text{AccE}}_{a}\right)}^{2}/48$ where 48 is the number of studied architectures. AccE_*a*_ and AccP_*a*_ are averages on 100 replicates, and denote respectively, for architecture *a*, the Empirical Accuracy and the Accuracy based on the chosen proxy.

	Fixed TRN matrix	TRN matrix varied
Theoretical accuracy	3.6710 × 10^−4^	4.204 × 10^−5^
Our proxy	5.9858 × 10^−4^	4.628 × 10^−4^
Proxy based on *M*_*e*1_	1.2643 × 10^−3^	1.228 × 10^−3^
Proxy based on *M*_*e*2_	1.207 × 10^−3^	1.157 × 10^−3^
Proxy based on *M*_*e*3_	1.1335 × 10^−3^	1.0669 × 10^−3^
Proxy based on *M*_*LJ*_	2.1906 × 10^−3^	1.474 × 10^−3^

#### Comparison between the effective number of segments and the quantity $n_{\text{TRN}}E({\left\|x_{n_{\text{TRN}}+1}^{\prime }X^{\prime }V^{-1}\right\|}^{2})$

According to our theoretical analysis, we should substitute the quantity $n_{\text{TRN}}E({\left\|x_{n_{\text{TRN}}+1}^{\prime }X^{\prime }V^{-1}\right\|}^{2})$ into [32]’s formula, instead of the number of independent loci, which is usually computed. Table 5 shows that $n_{\text{TRN}}E({\left\|x_{n_{\text{TRN}}+1}^{\prime }X^{\prime }V^{-1}\right\|}^{2})$ and *M*_*e*1_, *M*_*e*2_, *M*_*e*3_ and *M*_*LJ*_ are completely different quantities. Note that we focused on the configuration *n*_TRN_ = 500 (the case *n*_TRN_ = 1,000 is shown in S1 Table). In particular, we can see that $n_{\text{TRN}}E({\left\|x_{n_{\text{TRN}}+1}^{\prime }X^{\prime }V^{-1}\right\|}^{2})$ varied with the number of QTLs considered; this was not the case for other quantities. Table 6 shows analysis of the case where the TST population evolved during 30, 40, or 70 generations, where we kept a TRN population that evolved during 30 generations. This scenario is particularly realistic in plants, where a large number of generations can be obtained easily, and typically, the prediction model is not refitted with time. According to the table, for a given number of markers, the quantity $n_{\text{TRN}}E({\left\|x_{n_{\text{TRN}}+1}^{\prime }X^{\prime }V^{-1}\right\|}^{2})$ increased with the number of generations in the TST populations. In contrast, the usual quantities *M*_*e*1_, *M*_*e*2_, and *M*_*e*3_ could not capture the changes regarding the TST population because they depend only on the TRN population.

**Table 5:** Comparison among different estimators (*M*_*e*1_, *M*_*e*2_, *M*_*e*3_ and *M*_*LJ*_) of the number of effective loci and the quantity $n_{\text{TRN}}E({\left\|x_{n_{\text{TRN}}+1}^{\prime }X^{\prime }V^{-1}\right\|}^{2})$. For a given architecture, a mean was computed on 100 replicates (variance is shown in brackets) and the TRN incidence matrix did not vary across replicates (*n*_TRN_ = 500, λ is based on the heritability).

Nb QTLs	Nb generations	Nb Markers	$n_{\text{TRN}}E({\left\|x_{n_{\text{TRN}}+1}^{\prime }X^{\prime }V^{-1}\right\|}^{2})$	*M*_*LJ*_	*M*_*e*1_	*M*_*e*2_	*M*_*e*3_
2	30	100	47.16 (0.75)	50.13 (0.67)	11.96	14.02	16.92
		5,000	46.98 (0.58)	177.45 (4.72)	11.76	13.78	16.66
	50	100	53.63 (1.39)	59.73 (0.56)	17.71	20.43	24.12
		5,000	55.32 (0.61)	233.49 (7.36)	18.52	21.32	25.12
	70	100	51.26 (0.76)	64.39 (0.34)	22.68	25.93	30.27
		5,000	56.56 (0.67)	258.63 (7.89)	22.46	25.70	30.02
100	30	100	71.85 (2.40)	50.13 (0.70)	11.96	14.02	16.92
		5,000	74.83 (1.93)	177.45 (4.71)	11.76	13.78	16.66
	50	100	66.94 (2.31)	59.73 (0.50)	17.71	20.43	24.12
		5,000	70.66 (1.18)	233.49 (7.36)	18.52	21.32	25.12
	70	100	58.49 (1.06)	64.39 (0.34)	22.68	25.93	30.27
		5,000	66.68 (1.04)	258.63 (7.89)	22.46	25.70	30.02

**Table 6:** Comparison among different estimators (*M*_*e*1_, *M*_*e*2_, *M*_*e*3_ and *M*_*LJ*_) of the number of effective loci and the quantity $n_{\text{TRN}}E({\left\|x_{n_{\text{TRN}}+1}^{\prime }X^{\prime }V^{-1}\right\|}^{2})$ as a function of the number of generations during which the TST population evolved (TRN population is always based on 30 generations). For a given architecture, a mean was computed on 100 replicates (variance is shown in brackets) and the TRN incidence matrix did not vary across replicates (*n*_TRN_ = 500, and λ is based on the heritability).

Nb Markers	Nb generations for TST	$n_{\text{TRN}}E({\left\|x_{n_{\text{TRN}}+1}^{\prime }X^{\prime }V^{-1}\right\|}^{2})$	*M*_*LJ*_	*M*_*e*1_	*M*_*e*2_	*M*_*e*3_
100	30	47.16 (0.75)	50.13 (0.67)	11.96	14.02	16.92
	40	52.17 (0.93)	52.55 (0.84)	11.96	14.02	16.92
	70	58.17 (0.89)	55.95 (0.63)	11.96	14.02	16.92
5,000	30	46.98 (0.58)	177.45 (4.72)	11.76	13.78	16.66
	40	51.46 (0.60)	197.62 (5.21)	11.76	13.78	16.66
	70	52.86 (0.45)	229.45 (9.56)	11.76	13.78	16.66

### Real data

#### Accuracy

**Fig 6:**
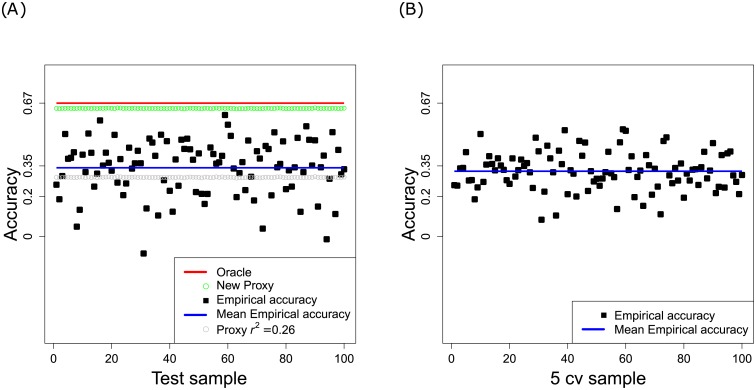
Empirical accuracy and proxies obtained for the perennial ryegrass dataset. (A) 90% TRN and 10% TST. (B) 5 fold cross-validation.

Fig 6A shows the Empirical accuracy estimated by means of the perennial ryegrass dataset, as a function of the TRN/TST samples under study. Readers will recall that 90% of the individuals were chosen randomly for the TRN set, and that the remaining 10% were considered TST individuals. According to the graph, there are large fluctuations between the different samples; this result points to the importance of a good match between TRN and TST sets (see [19, 38] regarding maize data, and [39] regarding simulated data).

Fig 6B illustrates results from 5-fold cross-validation. Readers can see that there is less variablity between the empirical accuracies than when the TRN incidence matrix is fixed for a given sample (see Fig 6A). This is in agreement with conclusions based on our simulation study because the cross-validation process can be viewed as an “unfixed” TRN matrix case, as opposed to the 90% TRN/10% TST process, which is a case of a fixed TRN matrix. As expected, the mean accuracy on all those samples was fairly similar in both analyses: it was estimated to be 0.35 for the 90% TRN/10% TST configuration, and 0.33 for the 5-fold cross-validation.

We computed the oracle accuracy (*h*), that is to say, the accuracy that would be achieved if the QTL locations and the QTL effects were known. The square root of the heritability was estimated to be 0.67 (${\hat{h}}^{2}=0.45$), because of the observed clones. This parameter was later evaluated for every TST sample, and only slight changes were observed (data not shown).

Next, we assessed perfomance of our new proxy. Although the proxy was reestimated for each TRN/TST configuration, we did not notice any fluctuations among the samples. The proxy should be regarded only as an upper bound: it is the optimal accuracy that can be achieved if the QTLs are in perfect LD with markers.

Because we suspected that the marker density was insufficient to cover the entire perennial ryegrass genome, our proxy was later adapted to the case of imperfect LD. In particular, we considered the work of [55], which is a generalization of [32]. Those authors assumed a constant LD *r*^2^ between each QTL and its associated marker, and they assumed the independence of each marker-QTL pair. In this context, our proxy can be rewritten as follows:

$$\rho_{\text{pLD}}=r^{2}\;h\;\sqrt{\frac{h^{2}/\left(1-h^{2}\right)}{E\left({\left\|x_{n_{\text{TRN}}+1}^{\prime }X^{\prime }V^{-1}\right\|}^{2}\right)+r^{2}\frac{h^{2}}{1-h^{2}}}}\;\text{.}$$ (8)

The *r*^2^ was estimated with the Pearson coefficient of correlation between pairs of SNPs belonging to the same scaffold. Due to the GBS method and the high level of polymorphism in perennial ryegrass (1 SNP/15 bp, see [64]), the distance between successive SNPs within a scaffold was not constant. Many SNPs were grouped within 150 bp because this value corresponded to the read length. The average distance between pairs of SNPs within the scaffolds separated by at least 150 bp was estimated to be 25,000 bp. In other words, blocks of several SNPs were separated on average by 25,000 bp. Assuming that these blocks are randomly distributed in the genome, the maximal distance between a QTL and a marker (SNP) is approximately 12,500 bp (25,000/2). As a consequence, the average *r*^2^ between pairs of SNPs separated by less than 12,500 bp, was computed: it was estimated to be 0.26 from the values of *r*^2^ as a function of genomic distance in base pairs (see S1 Fig). This value was later substituted into formula (8). Note that the method we used for calculating the quantity *r*^2^ is largely inspired by the work of [55].

According to Fig 6A, there is now a fairly good agreement between the proxy adapted for imperfect LD and the mean accuracy. This finding confirms our orginal supposition: the lack of markers must be responsible for the difference between our upper bound and the observed Empirical accuracy.

## Discussion

### General aspects

We present here a theoretical formula for the accuracy of GS. The theoretical advances were possible because we analyzed a causal model different from the prediction model (so-called marker-based model); this is usually not the case for investigators working on the mixed models (e.g. [34, 40]). Due to the recent progress in molecular biology, more and more genetic markers are becoming available, and it seems reasonable to assume that there are fewer QTLs than genetic markers in the genome. Recently, [53] incorporated this idea into the mixed-model framework. Nevertheless, those authors had to make approximations in order to obtain analytical formulas. In particular, those authors assumed that the TRN genomic relationships at causal loci were known, and then proposed to perform the regression of genomic relationships based on markers, on those based on causal loci. This idea was motivated by [65]. Although this concept seems interesting, it is not easy to implement and remains an empirical approach. In contrast, our proposed theoretical formula was derived rigorously, without approximations. The marker-based model chosen for our study, is the one corresponding to RRBLUP, also known as ridge regression. In other words, we considered the same high-dimensional prediction model and the same sparse causal model as those addressed in some recent statistical studies [66, 67].

With the help of our general formula (5), we are now able to quantify the influence of various parameters on the accuracy. The theoretical result depends on the QTL effects, QTL locations, TRN causal matrix, TRN incidence matrix, TST causal matrix and the TST incidence matrix. Although [68] highlighted the difficulty of decoding GBLUP, the final result is somewhat more complicated than what we expected and the results in the literature. For instance, according to our study, the average LD between markers is not proportional to the accuracy, contrarely to the results of [34]. In particular, we show that it is the quantity ***X***^**′**^ ***V***^**−1**^ ***Q*** that has an impact (that is to say, the LD between markers and QTLs in the TRN population with respect to the metric ***V***^**−1**^). This weighted LD can be viewed as an extension of the work of [69] where the authors introduced new LD measures corrected for population structure and relatedness. Moreover, according to our formula, the covariance between SNPs in the TST population affects the accuracy.

Our present study can be viewed as an answer to the analysis of [37], where the authors raised important questions regarding accuracy in GS. They compared 145 accuracy values extracted from 13 articles, either based on simulated data or real data. An analysis of variance model was fitted to the data, in order to test effects of 4 existing formulas and parameters, on the accuracy. The number of TRN individuals *n*_TRN_ and the effective number of segments *M*_*e*_ were found to have a strong influence on the accuracy. Besides, a “big formula effect” was observed, and those authors were unable to demonstrate superiority of one method to the others. One criticism voiced by those authors was that the different formulas in the literature did not take into account the relation between TRN and TST populations. Our theoretical formula now involves explicitly the link between the two populations.

The Theoretical accuracy depends on unknown parameters such as the QTL effects and their locations. It may be useful to first perform an association study on the TRN population in order to identify the QTLs. After that, the detected QTLs could be plugged into our theoretical formula (5), to approximate the accuracy. In the simulation study, since QTLs were perfectly known, such analysis was not considered relevant. Nonetheless, for the perennial ryegrass set, we could have explored this topic.

Most of the existing methods for computation of the accuracy are inspired by the work of [32] and [33]. In [33], the authors proposed to substitute the effective number of independent loci *M*_*e*_ into the original formula of [32]. Then, a large number of research groups elaborated on this concept, and proposed different ways of estimating *M*_*e*_, using either the effective population size (e.g. [34, 35]), or the number of independent tests [36].

Our theoretical analysis shows that plugging *M*_*e*_ into [32]’s formula is not the way to properly work with the high dimensional framework. We propose to use of another quantity, $n_{\text{TRN}}E({\left\|x_{n_{\text{TRN}}+1}^{\prime }X^{\prime }V^{-1}\right\|}^{2})$. We were able to show on simulated data that our corresponding proxy for the accuracy outperforms existing proxies. In the ryegrass dataset, however, most of the proxies studied yielded similar results because of the lack of markers to cover the entire genome.

An important question in GS is the choice of the TRN population. Various studies have shown sensitivity of the accuracy to relatedness between individuals. [38] focused on a population of maize and demonstrated that predictions are much more reliable when they are performed within families than across different families. Nevertheless, if we are willing to predict breeding values of a somewhat general TST population (not necessarily linked to the TRN population), [39] showed with the help of simulated data, that it is more advantageous to keep large variability in the TRN set.

Similarly, [19] proposed to merge populations belonging to different groups. Similar conclusions are also present in studies on sugar beets [25] and oats [70]. If we assume a limited budget (and as a consequence, a fixed number of TRN individuals), then an interesting finding in our study is the following: by minimizing the quantity $E({\left\|x_{n_{\text{TRN}}+1}^{\prime }X^{\prime }V^{-1}\right\|}^{2})$, we can choose the optimal TRN and TST sets, that maximize the accuracy. This protocol is an alternative to the “CDmean” method proposed by [40] that is based on the coefficient of determination (CD) to optimize the calibration sets. However, CDmean has a shortcoming: it does not differentiate the causal model from prediction model. In that sense, our approach to choosing the TRN and TST sets is expected to be more reliable than the one from [40]. This is a topic for future research. Concerning the computational burden, the Theoretical accuracy requires the inversion of a *n*_TRN_ × *n*_TRN_ matrix (complexity of $O(n_{\text{TRN}}^{3})$).

### GS in perennial ryegrass

In forage grasses, traits related to leaf growth (e.g., leaf length, plant height, and leaf elongation rate measured on spaced plants) are correlated with forage productivity measured in a dense canopy (i.e., sward; regarding short-term intake by cows and plant survival in a sward, see [71–73]). In perennial ryegrass, these traits represent a complex genetic architecture: many genes are involved in the overall variability. The corresponding heritability can be either medium (0.4) or high (0.7) [59, 74]. In this case, marker-assisted selection can detect only a small part of the genetic variation and very quickly becomes inefficient after fixation of the largest QTLs. In contrast, GS, which analyzes all the markers simultaneously, is potentially fruitful. Another interesting characteristic of GS is that it can reduce costs of phenotyping. In perennial ryegrass, the phenotyping of traits related to forage productivity is expensive and requires 2 or 3 years [75]. LD decreases rapidly with *r*, generally below 0.2 after less than 1kb [64].

In our study, we wanted to evaluate the accuracy of GS in a specific population created from elite material with a narrow genetic base (8 genotypes intercrossed during three generations). In this specific population, LD was expected to decrease slowly due to relatedness. In that case, we thought that a relatively small number of markers would be sufficient to cover the genome if the LD was large enough. According to our study, the mean accuracy is approximately 0.35. This value is similar to the results obtained on rice [27] and wheat [76]. The estimated accuracy was below that of our suggested proxy, which acted as an upper bound of the accuracy. Contrary to our original thoughts, the relatedness does not help to increase the accuracy and to compensate for the lack of markers. Indeed, our theoretical analysis shows that the accuracy depends on a weighted LD, ***X***^**′**^ ***V***^**−1**^ ***Q***, which can be viewed as LD corrected for the relatedness. On the other hand, GBS can also be considered as limiting factor for the accuracy. Although 20,000 markers were obtained by GBS, the number of independent markers is actually much smaller (approximately 4,500). Recall that a large number of markers is required to capture the genetic variability from many QTLs in the genome of perennial ryegrass. Finally, the accuracy was found to be strongly affected by the configuration of TRN and TST sets: the same phenomenon was observed in other studies (e.g., on oats [70]).

For all these reasons, in order to improve the accuracy of GS in perennial ryegrass, we propose to perform denser genotyping, and to select individuals to be phenotyped by minimizing the quantity $E({\left\|x_{n_{\text{TRN}}+1}^{\prime }X^{\prime }V^{-1}\right\|}^{2})$, according to our theoretical analysis. Fig 6A) shows that our suggested proxy was not very sensitive to the choice of the TRN set; we expect that an increase in the marker density will introduce some variability and help to choose the optimal TRN set.

## Supporting Information

S1 Text It includes the mathematical proof of the various formulas introduced in the section Materials and Methods. (PDF)

S2 Text Explanation of how to estimate *N*_*e*_ using the Hill and Weirr formula [54], and the LD computed between all SNPs. (PDF)

S1 Table This table is similar to Table 5 dealing with the case *n*_TRN_ = 1,000. (PDF)

S1 Fig LD, measured with *r*^2^, computed for the perennial ryegrass dataset, and associated with each pair of SNPs within scaffolds. (PDF)
