# Supplementary material for: On the Accuracy of Genomic Selection
Source: PLoS One. 2016 Jun 20;11(6):e0156086. doi: 10.1371/journal.pone.0156086 (PMC4913905; doi:10.1371/journal.pone.0156086)
Supplement: S1 Text — (PDF) [file pone.0156086.s001.pdf]

S1 Text. Supplementary material for the manuscript "On the accuracy of genomic selection"

1. Proof of the main result

In what follows, the quantities $\boldsymbol{X}$ and $\boldsymbol{Q}$ are supposed to be known. In other words, all the results will be conditional on $\boldsymbol{X}$ and $\boldsymbol{Q}$. Recall that $\boldsymbol{\theta}$ is fixed, and also that $\boldsymbol{q_{n_{\mathrm{TRN}}+1}}$ and $\boldsymbol{x_{n_{\mathrm{TRN}}+1}}$ are considered random.

Using the causal model, we have

$$\begin{aligned}\mathrm{Cov}\left(\hat{Y}_{n_{\mathrm{TRN}}+1},\ Y_{n_{\mathrm{TRN}}+1}\right) &= \mathrm{Cov}\left(\boldsymbol{x'_{n_{\mathrm{TRN}}+1}}\boldsymbol{X'V^{-1}Y},\ \boldsymbol{q'_{n_{\mathrm{TRN}}+1}}\boldsymbol{\theta} + e_{n_{\mathrm{TRN}}+1}\right)\\ &= \mathrm{Cov}\left(\boldsymbol{x'_{n_{\mathrm{TRN}}+1}}\boldsymbol{X'V^{-1}Q\theta},\ \boldsymbol{q'_{n_{\mathrm{TRN}}+1}}\boldsymbol{\theta}\right)\ .\end{aligned}$$

Besides, since $\boldsymbol{x_{n_{\mathrm{TRN}}+1}}$ and $\boldsymbol{q_{n_{\mathrm{TRN}}+1}}$ are centered, we have

$$\mathrm{Cov}\left(\hat{Y}_{n_{\mathrm{TRN}}+1},\ Y_{n_{\mathrm{TRN}}+1}\right) = \mathbb{E}\left(\boldsymbol{x'_{n_{\mathrm{TRN}}+1}}\boldsymbol{X'V^{-1}Q\theta q'_{n_{\mathrm{TRN}}+1}\theta}\right)\ . \qquad (1)$$

Let us now focus on the terms present at the denominator, that is to say $\mathrm{Var}\left(Y_{n_{\mathrm{TRN}}+1}\right)$ and $\mathrm{Var}\left(\hat{Y}_{n_{\mathrm{TRN}}+1}\right)$. By definition,

$$\mathrm{Var}\left(Y_{n_{\mathrm{TRN}}+1}\right) = \sigma_G^2\ +\ \sigma_e^2 \quad \text{where} \quad \sigma_G^2 = \boldsymbol{\theta'}\mathrm{Var}\left(\boldsymbol{q_{n_{\mathrm{TRN}}+1}}\right)\boldsymbol{\theta}\ . \qquad (2)$$

Besides, we have the relationship

$$\mathrm{Var}\left(\hat{Y}_{n_{\mathrm{TRN}}+1}\right) = \mathbb{E}\left(\mathrm{Var}\left(\hat{Y}_{n_{\mathrm{TRN}}+1} \mid \boldsymbol{x_{n_{\mathrm{TRN}}+1}}\right)\right) + \mathrm{Var}\left(\mathbb{E}\left(\hat{Y}_{n_{\mathrm{TRN}}+1} \mid \boldsymbol{x_{n_{\mathrm{TRN}}+1}}\right)\right)\ . \qquad (3)$$

To begin with, let us compute the quantity $\mathbb{E}\left(\mathrm{Var}\left(\hat{Y}_{n_{\mathrm{TRN}}+1} \mid \boldsymbol{x_{n_{\mathrm{TRN}}+1}}\right)\right)$. We have

$$\mathrm{Var}\left(\hat{Y}_{n_{\mathrm{TRN}}+1} \mid \boldsymbol{x_{n_{\mathrm{TRN}}+1}}\right) = \mathbb{E}\left(\hat{Y}^2_{n_{\mathrm{TRN}}+1} \mid \boldsymbol{x_{n_{\mathrm{TRN}}+1}}\right) - \left(\boldsymbol{x'_{n_{\mathrm{TRN}}+1}}\boldsymbol{X'V^{-1}Q\theta}\right)^2$$

and

$$\begin{aligned}\mathbb{E}\left(\hat{Y}^2_{n_{\mathrm{TRN}}+1} \mid \boldsymbol{x_{n_{\mathrm{TRN}}+1}}\right) &= \mathbb{E}\left(\left(\boldsymbol{x'_{n_{\mathrm{TRN}}+1}}\boldsymbol{X'V^{-1}Q\theta}\ +\ \boldsymbol{x'_{n_{\mathrm{TRN}}+1}}\boldsymbol{X'V^{-1}e}\right)^2 \mid \boldsymbol{x_{n_{\mathrm{TRN}}+1}}\right)\\ &= \mathbb{E}\left(\left(\boldsymbol{x'_{n_{\mathrm{TRN}}+1}}\boldsymbol{X'V^{-1}Q\theta}\right)^2 \mid \boldsymbol{x_{n_{\mathrm{TRN}}+1}}\right)\ +\ \mathbb{E}\left(\left(\boldsymbol{x'_{n_{\mathrm{TRN}}+1}}\boldsymbol{X'V^{-1}e}\right)^2 \mid \boldsymbol{x_{n_{\mathrm{TRN}}+1}}\right)\\ &= \left(\boldsymbol{x'_{n_{\mathrm{TRN}}+1}}\boldsymbol{X'V^{-1}Q\theta}\right)^2\ +\ \sigma_e^2\ \left\|\boldsymbol{x'_{n_{\mathrm{TRN}}+1}}\boldsymbol{X'V^{-1}}\right\|^2\end{aligned}$$

where $\|.\|$ is the L^2 norm. As a result,

$$\mathbb{E}\left(\mathrm{Var}\left(\hat{Y}_{n_{\mathrm{TRN}}+1} \mid \boldsymbol{x_{n_{\mathrm{TRN}}+1}}\right)\right) = \sigma_e^2\ \mathbb{E}\left(\left\|\boldsymbol{x'_{n_{\mathrm{TRN}}+1}}\boldsymbol{X'V^{-1}}\right\|^2\right)\ .$$

On the other hand, the second term in formula (3) is

$$\begin{aligned}\mathrm{Var}\left(\mathbb{E}\left(\hat{Y}_{n_{\mathrm{TRN}}+1} \mid \boldsymbol{x}_{\boldsymbol{n}_{\mathrm{TRN}}+\boldsymbol{1}}\right)\right) &= \mathrm{Var}\left(\boldsymbol{x}'_{\boldsymbol{n}_{\mathrm{TRN}}+\boldsymbol{1}}\boldsymbol{X}'\boldsymbol{V}^{-\boldsymbol{1}}\boldsymbol{Q}\boldsymbol{\theta}\right)\\ &= \boldsymbol{\theta}'\boldsymbol{Q}'\boldsymbol{V}^{-\boldsymbol{1}}\boldsymbol{X}\mathrm{Var}\left(\boldsymbol{x}_{\boldsymbol{n}_{\mathrm{TRN}}+\boldsymbol{1}}\right)\boldsymbol{X}'\boldsymbol{V}^{-\boldsymbol{1}}\boldsymbol{Q}\boldsymbol{\theta}\end{aligned}$$

where $\mathrm{Var}\left(\boldsymbol{x}_{\boldsymbol{n}_{\mathrm{TRN}}+\boldsymbol{1}}\right)$ is the covariance matrix of size $p \times p$. Then,

$$\mathrm{Var}\left(\hat{Y}_{n_{\mathrm{TRN}}+1}\right) = \sigma_e^2\,\mathbb{E}\left(\left\|\boldsymbol{x}'_{\boldsymbol{n}_{\mathrm{TRN}}+\boldsymbol{1}}\boldsymbol{X}'\boldsymbol{V}^{-\boldsymbol{1}}\right\|^2\right) + \boldsymbol{\theta}'\boldsymbol{Q}'\boldsymbol{V}^{-\boldsymbol{1}}\boldsymbol{X}\mathrm{Var}\left(\boldsymbol{x}_{\boldsymbol{n}_{\mathrm{TRN}}+\boldsymbol{1}}\right)\boldsymbol{X}'\boldsymbol{V}^{-\boldsymbol{1}}\boldsymbol{Q}\boldsymbol{\theta}\,. \tag{4}$$

To conclude, according to formulae (1), (2), (4), the accuracy ρ_{RR} satisfies the following expression

$$\rho_{\mathrm{RR}} = \frac{\mathbb{E}\left(\boldsymbol{x}'_{\boldsymbol{n}_{\mathrm{TRN}}+\boldsymbol{1}}\boldsymbol{X}'\boldsymbol{V}^{-\boldsymbol{1}}\boldsymbol{Q}\boldsymbol{\theta}\boldsymbol{q}'_{\boldsymbol{n}_{\mathrm{TRN}}+\boldsymbol{1}}\boldsymbol{\theta}\right)}{\left(\sigma_e^2\,\mathbb{E}\left(\left\|\boldsymbol{x}'_{\boldsymbol{n}_{\mathrm{TRN}}+\boldsymbol{1}}\boldsymbol{X}'\boldsymbol{V}^{-\boldsymbol{1}}\right\|^2\right) + \boldsymbol{\theta}'\boldsymbol{Q}'\boldsymbol{V}^{-\boldsymbol{1}}\boldsymbol{X}\mathrm{Var}\left(\boldsymbol{x}_{\boldsymbol{n}_{\mathrm{TRN}}+\boldsymbol{1}}\right)\boldsymbol{X}'\boldsymbol{V}^{-\boldsymbol{1}}\boldsymbol{Q}\boldsymbol{\theta}\right)^{1/2}\left(\sigma_G^2+\sigma_e^2\right)^{1/2}}\,. \tag{5}$$

Note that the formula can be rewritten:

$$\rho_{\mathrm{RR}} = \frac{\boldsymbol{\theta}'\,\mathbb{E}\left(\boldsymbol{q}_{\boldsymbol{n}_{\mathrm{TRN}}+\boldsymbol{1}}\boldsymbol{x}'_{\boldsymbol{n}_{\mathrm{TRN}}+\boldsymbol{1}}\right)\boldsymbol{X}'\boldsymbol{V}^{-\boldsymbol{1}}\boldsymbol{Q}\boldsymbol{\theta}}{\left(\sigma_e^2\,\mathbb{E}\left(\left\|\boldsymbol{x}'_{\boldsymbol{n}_{\mathrm{TRN}}+\boldsymbol{1}}\boldsymbol{X}'\boldsymbol{V}^{-\boldsymbol{1}}\right\|^2\right) + \boldsymbol{\theta}'\boldsymbol{Q}'\boldsymbol{V}^{-\boldsymbol{1}}\boldsymbol{X}\mathrm{Var}\left(\boldsymbol{x}_{\boldsymbol{n}_{\mathrm{TRN}}+\boldsymbol{1}}\right)\boldsymbol{X}'\boldsymbol{V}^{-\boldsymbol{1}}\boldsymbol{Q}\boldsymbol{\theta}\right)^{1/2}\left(\sigma_G^2+\sigma_e^2\right)^{1/2}}\,.$$

2. A new proxy (QTLs in perfect LD with some markers)

Let us assume that we have the relationship

$$\boldsymbol{x}'_{\boldsymbol{n}_{\mathrm{TRN}}+\boldsymbol{1}}\boldsymbol{X}'\boldsymbol{V}^{-\boldsymbol{1}}\boldsymbol{Q}\boldsymbol{\theta} = \boldsymbol{q}'_{\boldsymbol{n}_{\mathrm{TRN}}+\boldsymbol{1}}\boldsymbol{\theta}\,.$$

Let us consider the different terms present in the general formula (5). First, we have

$$\mathbb{E}\left(\boldsymbol{x}'_{\boldsymbol{n}_{\mathrm{TRN}}+\boldsymbol{1}}\boldsymbol{X}'\boldsymbol{V}^{-\boldsymbol{1}}\boldsymbol{Q}\boldsymbol{\theta}\boldsymbol{q}'_{\boldsymbol{n}_{\mathrm{TRN}}+\boldsymbol{1}}\boldsymbol{\theta}\right) = \mathbb{E}\left(\boldsymbol{q}'_{\boldsymbol{n}_{\mathrm{TRN}}+\boldsymbol{1}}\boldsymbol{\theta}\boldsymbol{q}'_{\boldsymbol{n}_{\mathrm{TRN}}+\boldsymbol{1}}\boldsymbol{\theta}\right) = \sigma_G^2\,.$$

Besides, since

$$\mathrm{Var}\left(\mathbb{E}\left(\hat{Y}_{n_{\mathrm{TRN}}+1} \mid \boldsymbol{x}_{\boldsymbol{n}_{\mathrm{TRN}}+\boldsymbol{1}}\right)\right) = \mathrm{Var}\left(\boldsymbol{x}'_{\boldsymbol{n}_{\mathrm{TRN}}+\boldsymbol{1}}\boldsymbol{X}'\boldsymbol{V}^{-\boldsymbol{1}}\boldsymbol{Q}\boldsymbol{\theta}\right) = \mathrm{Var}\left(\boldsymbol{q}'_{\boldsymbol{n}_{\mathrm{TRN}}+\boldsymbol{1}}\boldsymbol{\theta}\right) = \sigma_G^2\,,$$

we have

$$\mathrm{Var}\left(\hat{Y}_{n_{\mathrm{TRN}}+1}\right) = \sigma_e^2\,\mathbb{E}\left(\left\|\boldsymbol{x}'_{\boldsymbol{n}_{\mathrm{TRN}}+\boldsymbol{1}}\boldsymbol{X}'\boldsymbol{V}^{-\boldsymbol{1}}\right\|^2\right) + \sigma_G^2\,.$$

Then, the accuracy becomes,

$$\rho_{\mathrm{pLD}} = \frac{\sigma_G^2}{\left(\sigma_e^2\,\mathbb{E}\left(\left\|\boldsymbol{x}'_{\boldsymbol{n}_{\mathrm{TRN}}+\boldsymbol{1}}\boldsymbol{X}'\boldsymbol{V}^{-\boldsymbol{1}}\right\|^2\right) + \sigma_G^2\right)^{1/2}\left(\sigma_G^2+\sigma_e^2\right)^{1/2}}\,.$$

To conclude, since $\frac{\sigma_G^2}{\sigma_e^2} = \frac{h^2}{1-h^2}$, we obtain the final result

$$\rho_{\text{pLD}} = h \sqrt{\frac{h^2/(1-h^2)}{\mathbb{E}\left(\left\|\boldsymbol{x}'_{\boldsymbol{n}_{\text{TRN}}+\mathbf{1}}\boldsymbol{X}'\boldsymbol{V}^{-\mathbf{1}}\right\|^2\right) + \frac{h^2}{1-h^2}}} \ .$$

3. Link with the previous work of Daetwyler et al. [2008]

Estimators computed from Ridge Regression are equal to OLS estimators if λ is set to zero (see for instance Fan and Lv [2008]). So, by setting $\lambda = 0$, we obtain the prediction

$$\hat{\boldsymbol{Y}}_{\boldsymbol{n}_{\text{TRN}}+\mathbf{1}} = \boldsymbol{q}'_{\boldsymbol{n}_{\text{TRN}}+\mathbf{1}} \left(\boldsymbol{Q}'\boldsymbol{Q}\right)^{-\mathbf{1}} \boldsymbol{Q}'\boldsymbol{Y} \ \ .$$

Having replaced the terms $\boldsymbol{X}'\boldsymbol{V}^{-\mathbf{1}}$ by $(\boldsymbol{Q}'\boldsymbol{Q})^{-\mathbf{1}}\boldsymbol{Q}'$ and $\boldsymbol{x}_{\boldsymbol{n}_{\text{TRN}}+\mathbf{1}}$ by $\boldsymbol{q}_{\boldsymbol{n}_{\text{TRN}}+\mathbf{1}}$ in our general formula (5), the accuracy becomes

$$\begin{aligned}
\rho &= \frac{\mathbb{E}\left(\boldsymbol{q}'_{\boldsymbol{n}_{\text{TRN}}+\mathbf{1}}(\boldsymbol{Q}'\boldsymbol{Q})^{-\mathbf{1}}\boldsymbol{Q}'\boldsymbol{Q}\boldsymbol{\theta}\boldsymbol{q}'_{\boldsymbol{n}_{\text{TRN}}+\mathbf{1}}\boldsymbol{\theta}\right)}{\left(\sigma_e^2\mathbb{E}\left(\left\|\boldsymbol{q}'_{\boldsymbol{n}_{\text{TRN}}+\mathbf{1}}(\boldsymbol{Q}'\boldsymbol{Q})^{-\mathbf{1}}\boldsymbol{Q}'\right\|^2\right) + \boldsymbol{\theta}'\boldsymbol{Q}'\boldsymbol{Q}(\boldsymbol{Q}'\boldsymbol{Q})^{-\mathbf{1}}\text{Var}\left(\boldsymbol{q}_{\boldsymbol{n}_{\text{TRN}}+\mathbf{1}}\right)(\boldsymbol{Q}'\boldsymbol{Q})^{-\mathbf{1}}\boldsymbol{Q}'\boldsymbol{Q}\boldsymbol{\theta}\right)^{1/2}\left(\sigma_G^2+\sigma_e^2\right)^{1/2}} \\
&= \frac{\mathbb{E}\left(\boldsymbol{q}'_{\boldsymbol{n}_{\text{TRN}}+\mathbf{1}}\boldsymbol{\theta}\boldsymbol{q}'_{\boldsymbol{n}_{\text{TRN}}+\mathbf{1}}\boldsymbol{\theta}\right)}{\left(\sigma_e^2\mathbb{E}\left(\left\|\boldsymbol{q}'_{\boldsymbol{n}_{\text{TRN}}+\mathbf{1}}(\boldsymbol{Q}'\boldsymbol{Q})^{-\mathbf{1}}\boldsymbol{Q}'\right\|^2\right) + \boldsymbol{\theta}'\text{Var}\left(\boldsymbol{q}_{\boldsymbol{n}_{\text{TRN}}+\mathbf{1}}\right)\boldsymbol{\theta}\right)^{1/2}\left(\sigma_G^2+\sigma_e^2\right)^{1/2}} \\
&= \frac{\sigma_G^2}{\left(\sigma_e^2\mathbb{E}\left(\left\|\boldsymbol{q}'_{\boldsymbol{n}_{\text{TRN}}+\mathbf{1}}(\boldsymbol{Q}'\boldsymbol{Q})^{-\mathbf{1}}\boldsymbol{Q}'\right\|^2\right) + \sigma_G^2\right)^{1/2}\left(\sigma_G^2+\sigma_e^2\right)^{1/2}} \\
&= \frac{h\ \sigma_G}{\left(\sigma_e^2\mathbb{E}\left(\left\|\boldsymbol{q}'_{\boldsymbol{n}_{\text{TRN}}+\mathbf{1}}(\boldsymbol{Q}'\boldsymbol{Q})^{-\mathbf{1}}\boldsymbol{Q}'\right\|^2\right) + \sigma_G^2\right)^{1/2}}.
\end{aligned}$$

To finish, we use that (proof given in next section)

$$\mathbb{E}\left(\left\|\boldsymbol{q}'_{\boldsymbol{n}_{\text{TRN}}+\mathbf{1}}(\boldsymbol{Q}'\boldsymbol{Q})^{-\mathbf{1}}\boldsymbol{Q}'\right\|^2\right) \approx \frac{C}{n_{\text{TRN}}} \ ,$$

so the phenotypic accuracy and the genotypic accuracy are the following

$$\rho = h\sqrt{\frac{h^2/(1-h^2)}{\frac{C}{n_{\text{TRN}}} + \frac{h^2}{1-h^2}}} \ , \tilde{\rho} = \sqrt{\frac{h^2/(1-h^2)}{\frac{C}{n_{\text{TRN}}} + \frac{h^2}{1-h^2}}} \ \ .$$

In Daetwyler et al. [2008], the authors consider the case $\sigma_G^2 + \sigma_e^2 = 1$. As a result,

$$\rho = \frac{h^2}{\sqrt{\sigma_e^2\ \frac{C}{n_{\text{TRN}}}\ +\ h^2}} \quad , \quad \tilde{\rho} = \frac{h}{\sqrt{\sigma_e^2\ \frac{C}{n_{\text{TRN}}}\ +\ h^2}} \ . \qquad (6)$$

Besides, they use the approximation $\sigma_e^2 \approx 1$. Using this approximation in (6) and simplifying by h, we obtain

$$\rho = \frac{h}{\sqrt{\frac{1}{\eta h^2} + 1}} \quad , \quad \tilde{\rho} = \sqrt{\frac{h^2 \eta}{1 + h^2 \eta}} \quad \text{where} \quad \eta = n_{\mathrm{TRN}}/C \; .$$

We can notice that this expression of $\tilde{\rho}$ is the same as the one presented in formula (1) of Daetwyler et al. [2008]. In the same way, the expression for ρ is the same as the one given at the end of Appendix A of Visscher et al. (2010), except that the focus was on the quantity ρ^2.

Later, in their paper, Daetwyler et al. [2008] relaxed the assumption $\sigma_e^2 \approx 1$, and studied another approximation: $\sigma_e^2 \approx (1 - h^2) + h^2(1 - \tilde{\rho}^2)$. Using this new approximation in formula (6), we obtain

$$\tilde{\rho}^2 = \frac{h^2 \eta}{(1 - h^2 \tilde{\rho}^2) + h^2 \eta}$$

which is the same quantity as presented in formula (1) of Appendix S1 of Daetwyler et al. [2008].

4. Proof of $\mathbb{E}\left(\left\|\boldsymbol{q}'_{n_{\mathrm{TRN}}+1}(\boldsymbol{Q}'\boldsymbol{Q})^{-1}\boldsymbol{Q}'\right\|^2\right) \approx C/n_{\mathrm{TRN}}$

Recall that Daetwyler et al. [2008] suppose that the matrix $(\boldsymbol{Q}'\boldsymbol{Q})^{-1}$ is diagonal and then,

$$(\boldsymbol{Q}'\boldsymbol{Q})^{-1}_{j,j} = \left(\sum_{i=1}^{n_{\mathrm{TRN}}} Q_{i,j}^2\right)^{-1} \; .$$

Let d_1, ..., d_C denote the quantities such as

$$(\boldsymbol{Q}'\boldsymbol{Q})_{j,j} = d_j \quad (j = 1, \cdots, C) \; .$$

Let $q_{n_{\mathrm{TRN}}+1,j}$ denote the genotype of the TST individual at the jth QTL. We have the relationship

$$\begin{aligned}\left\|\boldsymbol{q}'_{n_{\mathrm{TRN}}+1}(\boldsymbol{Q}'\boldsymbol{Q})^{-1}\boldsymbol{Q}'\right\|^2 &= \sum_{j=1}^{C} \frac{(q_{n_{\mathrm{TRN}}+1,j})^2}{d_j} \\ &+ 2 \sum_{j=1}^{C-1} \sum_{j'=j+1}^{C} \frac{q_{n_{\mathrm{TRN}}+1,j} \; q_{n_{\mathrm{TRN}}+1,j'} \; \sum_{i=1}^{n_{\mathrm{TRN}}} (Q_{i,j} Q_{i,j'})}{d_j \; d_{j'}} \; .\end{aligned}$$

Since the QTLs are assumed to be in linkage equilibrium, we have

$$\forall j \neq j', \; \mathbb{E}\left(q_{n_{\mathrm{TRN}}+1,j} \; q_{n_{\mathrm{TRN}}+1,j'}\right) = 0 \; .$$

Recall that computations are conditional on $\boldsymbol{Q}$. As a consequence,

$$\mathbb{E}\left(2\sum_{j=1}^{C-1}\sum_{j'=j+1}^{C}\frac{q_{n_{\mathrm{TRN}}+1,j}\ q_{n_{\mathrm{TRN}}+1,j'}\ \sum_{i=1}^{n_{\mathrm{TRN}}}\left(Q_{i,j}Q_{i,j'}\right)}{d_j\ d_{j'}}\right)=0\ .$$

Then,

$$\mathbb{E}\left(\left\|\boldsymbol{q}'_{\boldsymbol{n}_{\mathrm{TRN}}+\mathbf{1}}(\boldsymbol{Q}'\boldsymbol{Q})^{-\mathbf{1}}\boldsymbol{Q}'\right\|^2\right)=\sum_{j=1}^{C}\frac{\mathbb{E}\left(\left(q_{n_{\mathrm{TRN}}+1,j}\right)^2\right)}{d_j}\ .$$

Finally, the authors use the approximation, $d_j \approx n_{\mathrm{TRN}}\mathbb{E}\left(\left(Q_{n_{\mathrm{TRN}},j}\right)^2\right)$ suitable when n_{TRN} is large. Besides, they assume that the TST and TRN samples come from the same population. In this context, $Q_{1,j},\ldots,Q_{n_{\mathrm{TRN}},j},q_{n_{\mathrm{TRN}}+1,j}$ are i.i.d. variables, and we have the relationship

$$\begin{aligned}\mathbb{E}\left(\sum_{j=1}^{C}\frac{\left(q_{n_{\mathrm{TRN}}+1,j}\right)^2}{d_j}\right)&\approx\sum_{j=1}^{C}\frac{\mathbb{E}\left(\left(q_{n_{\mathrm{TRN}}+1,j}\right)^2\right)}{n_{\mathrm{TRN}}\ \mathbb{E}\left(\left(Q_{n_{\mathrm{TRN}},j}\right)^2\right)}\\&\approx C/n_{\mathrm{TRN}}\ .\end{aligned}$$

As a result,

$$\mathbb{E}\left(\left\|\boldsymbol{q}'_{\boldsymbol{n}_{\mathrm{TRN}}+\mathbf{1}}(\boldsymbol{Q}'\boldsymbol{Q})^{-\mathbf{1}}\boldsymbol{Q}'\right\|^2\right)\approx C/n_{\mathrm{TRN}}\ .$$

It concludes the proof.

References

Daetwyler, H.D., Villanueva, B., Woolliams, J.A. (2008) Accuracy of predicting the genetic risk of disease using a genome-wide approach. *PLoS One*, **3(10)**, e3395.

Daetwyler, H.D., Villanueva, B., Woolliams, J.A. (2010) The impact of genetic architecture on genome-wide evaluation methods. *Genetics*, **185(3)**, 1021-1031.

Visscher, P.M., Yang, J., Goddard, M.E. (2010) A commentary on "common SNPs explain a large proportion of the heritability for human height" by Yang et al.(2010). *Twin Research and Human Genetics*, **13(06)**, 517-524.

Fan, J., Lv, J. (2008) Sure independence screening for ultrahigh dimensional feature space. *Journal of the Royal Statistical Society Series B*, **70(5)**, 849-911.

Meuwissen, T.H., Hayes, B., Goddard, M.E. (2001) Prediction of total genetic value using genome-wide dense marker maps. *Genetics*, **157(4)**, 1819-1829.
