# Supplementary material for: On the Accuracy of Genomic Selection
Source: PLoS One. 2016 Jun 20;11(6):e0156086. doi: 10.1371/journal.pone.0156086 (PMC4913905; doi:10.1371/journal.pone.0156086)
Supplement: S2 Text — (PDF) [file pone.0156086.s002.pdf]

# S2 Text. Supplementary material for the manuscript "On the accuracy of genomic selection"

Let $c_{jj'}$ denote the recombination rate beween loci $j$ and $j'$ (Haldane model in this paper), $N_e$ the effective population size, $n_{\text{TRN}}$ the sample size and $r^2_{jj'}$ the squared Pearson correlation of genotypes at the two loci (LD). According to the formula p.77 of Hill and Weirr (1988), we have

$$\mathbb{E}\left(r^2_{jj'}\right) = g(\Gamma_{jj'})$$

where

$$g(\Gamma_{jj'}) = \frac{(10+\Gamma_{jj'})}{(2+\Gamma_{jj'})(11+\Gamma_{jj'})}\left(1+\frac{(3+\Gamma_{jj'})(12+12\Gamma_{jj'}+\Gamma^2_{jj'})}{n_{\text{TRN}}(2+\Gamma_{jj'})(11+\Gamma_{jj'})}\right) \quad ,$$

$$\Gamma_{jj'} = 4N_e\, c_{jj'} \quad .$$

Since we are only able to get an estimated LD between the two loci $j$ and $j'$, we can reasonably consider that

$$\hat{r}^2_{jj'} = g(\Gamma_{jj'}) \; + \varepsilon$$

where $\varepsilon$ denotes some noise. Then, the $N_e$ can be estimated in the following way,

$$\widehat{N_e} = \arg\min_{N_e} \sum \left\{\hat{r}^2_{jj'} - g(\Gamma_{jj'})\right\}^2$$

where the sum is over all the pairs of loci.

## References


Hill, WG and Weir, BS (1998) Variances and covariances of squared linkage disequilibria in finite population. *Theoretical population biology*, **33(1)**, 54-78.
