# Supplementary material for: On the Accuracy of Genomic Selection
Source: PLoS One. 2016 Jun 20;11(6):e0156086. doi: 10.1371/journal.pone.0156086 (PMC4913905; doi:10.1371/journal.pone.0156086)
Supplement: S1 Table — (PDF) [file pone.0156086.s003.pdf]

S1 Table. Supplementary material for the manuscript "On the accuracy of genomic selection"

Table 1: Comparison between different estimators (M_{e1}, M_{e2}, M_{e3} and M_{LJ}) of the number of effective loci and the quantity $n_{\text{TRN}}\mathbb{E}\left(\left\|\boldsymbol{x}'_{\boldsymbol{n}_{\text{TRN}}+\boldsymbol{1}}\boldsymbol{X}'\boldsymbol{V}^{-\boldsymbol{1}}\right\|^2\right)$. For a given architecture, a mean was computed on 100 replicates (variance is shown in brackets) and the TRN incidence matrix did not vary across replicates (n_{TRN} =1,000, λ is based on the heritability).

Nb QTLs	Nb generations	Nb Markers	$n_{\text{TRN}}\mathbb{E}\left(\left\|\boldsymbol{x}'_{\boldsymbol{n}_{\text{TRN}}+\boldsymbol{1}}\boldsymbol{X}'\boldsymbol{V}^{-\boldsymbol{1}}\right\|^2\right)$	M_{LJ}	M_{e1}	M_{e2}	M_{e3}
2	30	100	60.33 (1.53)	52.33 (0.40)	12.49	14.60	17.58
		5,000	75.40 (2.17)	231.2 (6.28)	11.36	13.34	16.16
	50	100	69.22 (1.55)	61.12 (0.15)	19.60	22.52	26.47
		5,000	77.95 (1.41)	280.81 (8.53)	14.09	16.40	19.60
	70	100	69.72 (0.73)	69.95 (0.11)	26.38	30.02	34.84
		5,000	90.25 (1.70)	331.94 (7.88)	21.17	24.26	28.41
100	30	100	88.25 (4.67)	52.33 (0.41)	12.49	14.60	17.58
		5,000	130.29 (7.35)	231.2 (6.28)	11.36	13.34	16.16
	50	100	85.80 (3.04)	61.12 (0.14)	19.60	22.52	26.47
		5,000	109.59 (3.47)	280.81 (8.53)	14.09	16.40	19.60
	70	100	82.38 (1.22)	69.95 (0.11)	26.38	30.02	34.84
		5,000	115.97 (3.33)	331.94 (7.88)	21.17	24.26	28.41
