# Supplementary material for: On the Accuracy of Genomic Selection
Source: PLoS One. 2016 Jun 20;11(6):e0156086. doi: 10.1371/journal.pone.0156086 (PMC4913905; doi:10.1371/journal.pone.0156086)
Supplement: S1 Fig — (PDF) [file pone.0156086.s004.pdf]

S1 Fig. Supplementary material for the manuscript “On the accuracy of genomic selection”

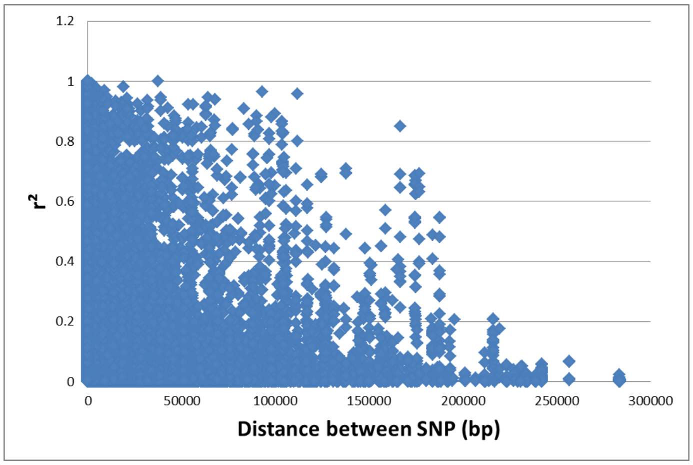

Fig 1: Linkage Disequilibrium (LD), measured with r^2, computed for the perennial ryegrass dataset, and associated with each pair of SNPs within scaffolds.
